# Grasping the big picture: impact analysis of screening tools for timely referral for device-aided therapies

**DOI:** 10.1007/s00702-024-02783-1

**Published:** 2024-07-15

**Authors:** H. R. Moes, H. S. Dafsari, W. H. Jost, N. Kovacs, Z. Pirtošek, T. Henriksen, C. Falup-Pecurariu, M. Minár, E. Buskens, T. van Laar

**Affiliations:** 1 Department of Neurology, University Medical Center Groningen, University of Groningen, Groningen, The Netherlands; 2 Department of Neurology, Faculty of Medicine and University Hospital Cologne, University of Cologne, Cologne, Germany; 3 Parkinson-Klinik Ortenau, Kreuzbergstr. 12‑16, Wolfach, 77709 Germany; 4 Department of Neurology, University of Pecs, Medical School, 48-as tér 1, Pecs, Hungary; 5 Department of Neurology, University Medical Center, Ljubljana, Slovenia; 6 Movement Disorder Clinic, University Hospital of Bispebjerg, Copenhagen, Denmark; 7 Department of Neurology, Faculty of Medicine, County Clinic Hospital, Faculty of Medicine, Transylvania University, Braşov, Romania; 8 Second Department of Neurology, Faculty of Medicine, Comenius University Bratislava, Bratislava, Slovakia; 9 Department of Epidemiology, University Medical Center Groningen, University of Groningen, Groningen, The Netherlands; 10 Department of Neurology, Faculty of Medicine, University of Ljubljana, Ljubljana, Slovenia

**Keywords:** Parkinson’s disease, Device-aided therapies, Screening tools, Impact analysis, Timely referral

## Abstract

Several screening tools are available to assist general neurologists in the timely identification of patients with advanced Parkinson’s disease (PD) who may be eligible for referral for a device-aided therapy (DAT). However, it should be noted that not all of these clinical decision rules have been developed and validated in a thorough and consistent manner. Furthermore, only a limited number of head-to-head comparisons have been performed. Available studies suggest that D-DATS has a higher positive predictive value and higher specificity than the 5-2-1 criteria, while the sensitivity of both screening tools is similar. However, unanswered questions remain regarding the validity of the decision rules, such as whether the diagnostic performance measures from validation studies are generalizable to other populations. Ultimately, the question is whether a screening tool will effectively and efficiently improve the quality of life of patients with PD. To address this key question, an impact analysis should be performed. The authors intend to set up a multinational cluster randomised controlled trial to compare the D-DATS and 5-2-1 criteria on the downstream consequences of implementing these screening tools, with a particular focus on the impact on disability and quality of life.

## Introduction

Parkinson’s disease (PD) is a common neurodegenerative disorder for which there is no cure (Bloem et al. 2021). The incidence and prevalence of PD are increasing, with an estimated global prevalence of 6.1 million patients in 2016 and a projected prevalence of 12.9 million patients in 2040 (Dorsey and Bloem 2018; Dorsey et al. 2018; Ben-Shlomo et al. 2024). Treatment for people with PD aims to reduce symptoms and improve functioning and quality of life (Foltynie et al. 2024). Pharmacologic treatment options for PD include oral and transdermal dopaminergic medications, with oral levodopa as the treatment of first choice (de Bie et al. 2020; Foltynie et al. 2024).

As PD progresses, the oral and transdermal treatments may become less effective and patients tend to develop motor complications, such as response fluctuations and dyskinesias (Armstrong and Okun 2020; Bloem et al. 2021). When motor complications can no longer be alleviated by optimizing the oral and transdermal treatment regimen, device-aided therapies (DAT) such as deep brain stimulation (DBS), continuous subcutaneous apomorphine injection (CSAI), levodopa/carbidopa intestinal gel infusion (LCIG) or levodopa/carbidopa/entacapone intestinal gel infusion (LECIG) may be considered (Deuschl et al. 2022; Fabbri et al. 2023).

Due to the gradual and variable progression of PD, marking the stage of advanced PD and timing the initiation of DAT are not straightforward (Odin et al. 2015; Antonini et al. 2018; Pirtošek et al. 2020). In addition, there is no universal test or measure to determine if and when a patient should be referred for DAT (Auffret et al. 2023). Furthermore, patient-related factors influence the decision-making process, including age, cognition, comorbidity, type and severity of motor and non-motor symptoms, response to medication, and preferences and expectations of both the patient and their partner or other caregiver (Antonini et al. 2018; Nijhuis et al. 2019; Auffret et al. 2023). Importantly, non-patient factors may influence considerations for initiating DAT, such as local availability of care (e.g., national regulatory approval of DAT types, regional availability of DAT types, resource capacity of specialist DAT centres) as well as financial aspects (e.g., cost of DAT, reimbursement issues) (Auffret et al. 2023; Borovečki et al. 2023; Moes et al. 2023b).

Worldwide, there is evidence of both under- and over-referral of PD patients for DAT, consistent with the observed significant regional differences in the use of DAT (Richter et al. 2019; Henriksen et al. 2020; Auffret et al. 2023). Under-referral has been observed in Poland and Romania (Szasz et al. 2021; Moes et al. 2023b), and may result in missed opportunities to provide an effective treatment option to patients who may benefit from it. In contrast, over-referral is evident from observations of DBS referrals in the Netherlands, Germany, and Russia (Wächter et al. 2011; Geraedts et al. 2019; Bril et al. 2021). Similarly, the personal experience of some of our authors suggests that a significant proportion of referrals are inappropriate, e.g. PD patients who do not have motor complications or treatment-resistant tremor, or PD patients in whom the oral medication could easily be further optimised (comment based on authors’ personal experience). These observations are consistent with studies showing that general neurologists lack competence in identifying patients who may be candidates for DAT (Lange et al. 2017; Moes et al. 2023c).

For final assessment of eligibility for DAT, PD patients should be referred to a specialised clinic where movement disorder specialists carry out a comprehensive eligibility assessment (Auffret et al. 2023). Several tools and criteria have been developed to assist general neurologists in the timely referral of patients with advanced PD to these specialised centres (Moes et al. 2023b). Such screening tools for timely referral aim to prevent patients from being referred for DAT either too early or too late (Table 1). Widespread implementation of an accurate tool could potentially also help reduce regional disparities in access to DAT (Moes et al. 2023b).

**Table 1:** Potential disadvantages for general neurologists and movement disorder specialists in either not using or indeed using screening tools for timely referral for device-aided therapies

Scenario	General neurologists	Movement disorders specialists
*Not using a screening tool*	- difficulties in identifying potential candidates for DAT; - hesitation or reluctance to refer a patient for DAT; - arbitrariness of referral; - differences in referral rates between neurologists and between regions.	- few patients eligible for DAT are referred in time; - patients referred late have already developed contraindications for DAT; - high numbers of inappropriate referrals may lead to waiting lists.
*Use of a screening tool with suboptimal screening performance (*e.g., low sensitivity and/or low specificity and low positive predictive value)	- time spent using the tool; - unrealistic expectations among patients and referrers, - low yield makes it unattractive to continue using the tool	- over-referral when positive predictive value is low: many patients are referred who are not (yet) eligible; - under-referral when sensitivity is low.
*Use of a high performance screening tool*	- time required to use the tool; - no other expected disadvantages for a tool with a high sensitivity, specificity and positive predictive value	- no disadvantages expected for a tool with high sensitivity, specificity and positive predictive value

DAT = device-aided therapy

**Fig. 1:**
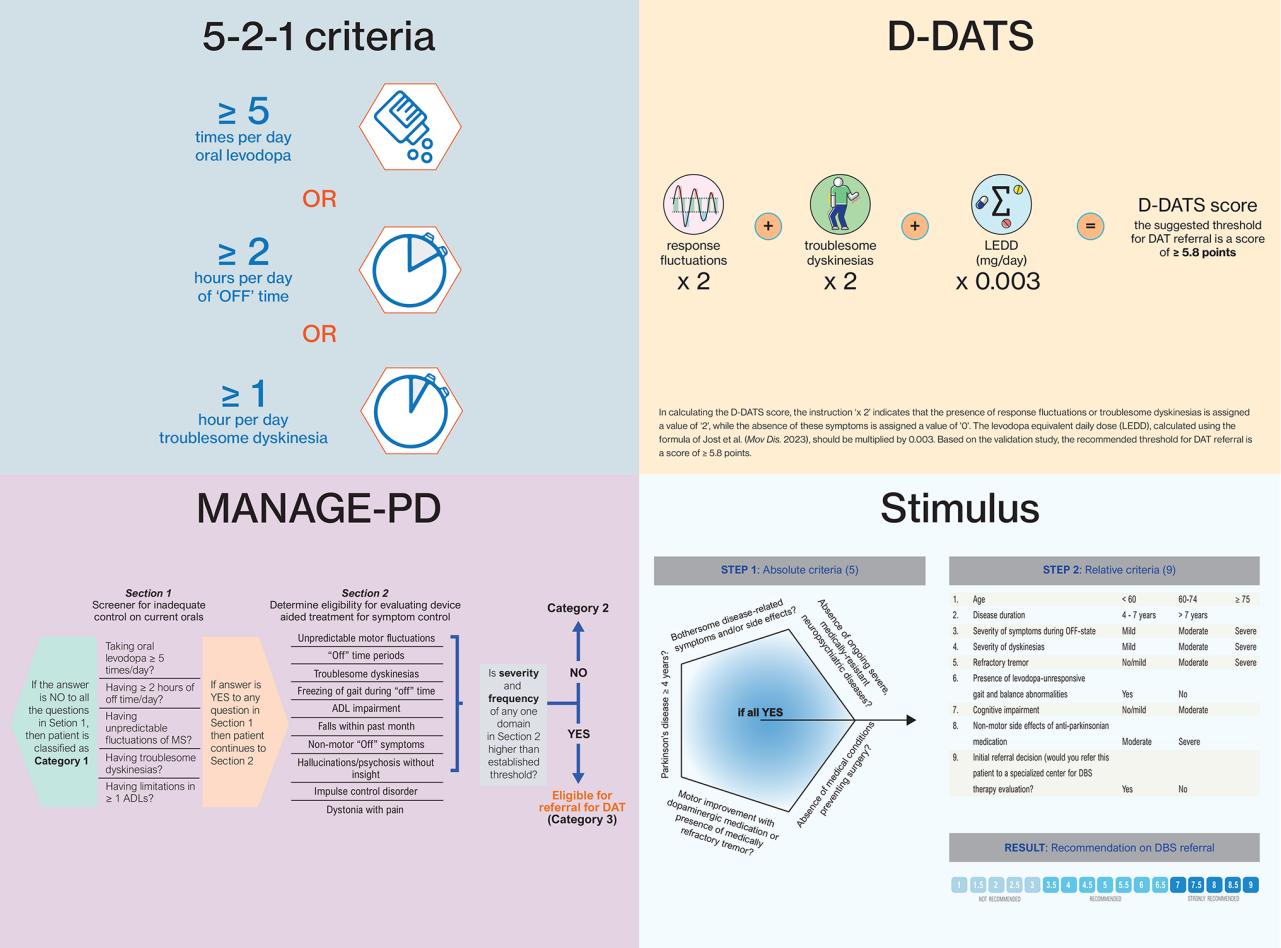
Graphical representation of four screening tools: the 5-2-1 criteria (Antonini et al. 2018), D-DATS (Moes et al. 2023a, d), MANAGE-PD (Antonini et al. 2021), and Stimulus (Moro et al. 2016). Figures have been adapted from the references listed. Specifically, the figure of MANAGE-PD has been adapted (i.e. Category 3 was specified as ‘eligible for referral for DAT’) and used under the terms of the Creative Commons Attribution License (CC BY; (https://creativecommons.org/licenses/by/4.0/)

Examples of available screening tools include the 5-2-1 criteria (Malaty et al. 2022), MANAGE-PD (Antonini et al. 2021), D-DATS (Moes et al. 2023a, d), and Stimulus (Moro et al. 2009, 2016) (Table 2; Fig. 1). In general, these tools include a combination of different patient and treatment characteristics, such as the duration and frequency of “off” periods and the amount of dopaminergic medication taken per day (Auffret et al. 2023; Moes et al. 2023b).

**Table 2:** Four examples of available tools to screen for advanced PD or eligibility for DAT referral. See also Fig. 1. A more comprehensive table of all available screening tools can be accessed elsewhere (Moes et al. 2023b)

Tool	Aim	Description	Limitations
*5-2-1 criteria*	Identify patients with advanced PD	≥ 5 doses of oral levodopa per day and/or ≥ 2 h of “off” time per day, and/ or ≥ 1 h of troublesome dyskinesia per day.	- Not specifically developed for identifying DAT eligibility. - Not developed according to TRIPOD guidelines. - Low positive predictive value.
*D-DATS*	Eligibility for referral for *any* DAT	Three-factor screening tool based on LEDD, response fluctuations, and troublesome dyskinesias.	- Not sensitive for treatment resistant tremor. - Development and validation in the Netherlands.
*MANAGE-PD*	Eligibility for referral for *any* DAT	Two sections. First section 5 questions. Second section 7 elements.	- Relatively time-consuming (many questions). - Not developed nor validated according to TRIPOD guidelines. - Not a parsimonious model.
*Stimulus*	Eligibility for referral for DBS	Two sections. First 5 questions on absolute criteria. Secondly 9 questions on relative criteria.	- Only useful for assessing eligibility for referral for DBS. - Updated Stimulus has not been validated.

DAT = device-aided therapy; DBS = deep brain stimulation; D-DATS = Dutch Device-Aided Therapy Screening*; LEDD = levodopa equivalent daily dose; MANAGE-PD = Making Informed Decisions to Aid Timely Management of Parkinson’s Disease; PD = Parkinson’s disease; TRIPOD = Transparent Reporting of a multivariable prediction model for Individual Prognosis or Diagnosis

* The adjective “Dutch” in D-DATS refers to the country in which the D-DATS tool was developed and validated, but it should be noted that the tool is in fact available in English

Although the available screening tools have received considerable attention in the international literature, limitations and criticisms have also been raised. For example, the MANAGE-PD was developed and validated using a suboptimal methodology (Antonini et al. 2021; Moes et al. 2022). In addition, the 5-2-1 criteria have a relatively low positive predictive value, meaning that many patients who score positive on the tool are not actually eligible for DAT referral (Moes et al. 2023b). This may lead to inappropriate referrals and an additional burden on the referral network.

Given the variety of screening tools for timely referral for DAT, there is a need to clarify which tool has the best performance in clinical practice. This position paper presents a perspective on answering this question, i.e. how to find the optimal screening tool for timely referral of PD patients for DAT while minimizing over-referral. The ultimate goal is to improve the quality of life of PD patients, not simply to increase the number of DAT users. To achieve this goal, we advocate conducting an impact analysis using a cluster randomised controlled trial.

## Under-referral and over-referral

In PD patients with motor complications for whom the oral and/or transdermal medication regimen cannot be further optimised, DATs contribute to improved motor and non-motor scores and quality of life (Dafsari et al. 2018; Bloem et al. 2021; Deuschl et al. 2022). Therefore, timely identification of patients who may be eligible for DAT is deemed important. In addition, delaying referral may result in the patient having already developed contraindications to one or more types of DAT, i.e. the optimal time to initiate DAT has passed.

Nonetheless, optimisation of the oral and transdermal medication regimen with adjuvant therapies (MAO-B inhibitors, dopamine agonists, COMT inhibitors and amantadine) remains key, as these optimisation steps are less invasive and less expensive than DATs (Stowe et al. 2010; Gray et al. 2022; Hauser et al. 2022; Sako et al. 2023). This is likely to be even more important in healthcare systems that face tighter budget constraints and therefore have fewer resources to offer DAT (Auffret et al. 2023; Chaudhuri and Batzu 2023).

It should also be noted that in patients for whom the treating physician considers DAT appropriate, up to 50% refuse referral or delay initiation of DAT at the time of discussion (Dinkelbach et al. 2017; Szász et al. 2019; Fasano et al. 2019; Moes et al. 2023d). Given this patient reluctance, timely identification of patients potentially eligible for DAT using a screening tool will not necessarily result in referral of all identified patients.

In short, it is important for general neurologists treating PD patients to recognise when a patient is eligible for referral to a hospital offering DAT (Pirtošek et al. 2020). There is, of course, a trade-off between very early recognition and late referral. Too early recognition can lead to over-referral and unnecessary burden on the referral system. Late referral may delay initiation of an effective treatment option to patients who could have already benefitted from it.

In the field of PD care and DAT, four groups of stakeholders may be identified: patients, healthcare providers, health insurance companies, and the pharmaceutical industry. When evaluating screening tools for DAT referral, each of these four groups is likely to prefer timely referral, given that DAT can be a cost-effective treatment that leads to significant health gains in adequately selected patients (Norlin et al. 2021). However, when considering the balance of desirability between high sensitivity (i.e. more false positives, hence over-referral) and high specificity (i.e. more false negatives, hence under-referral) of such tools, the four different groups may have different preferences (Table 3).

**Table 3:**
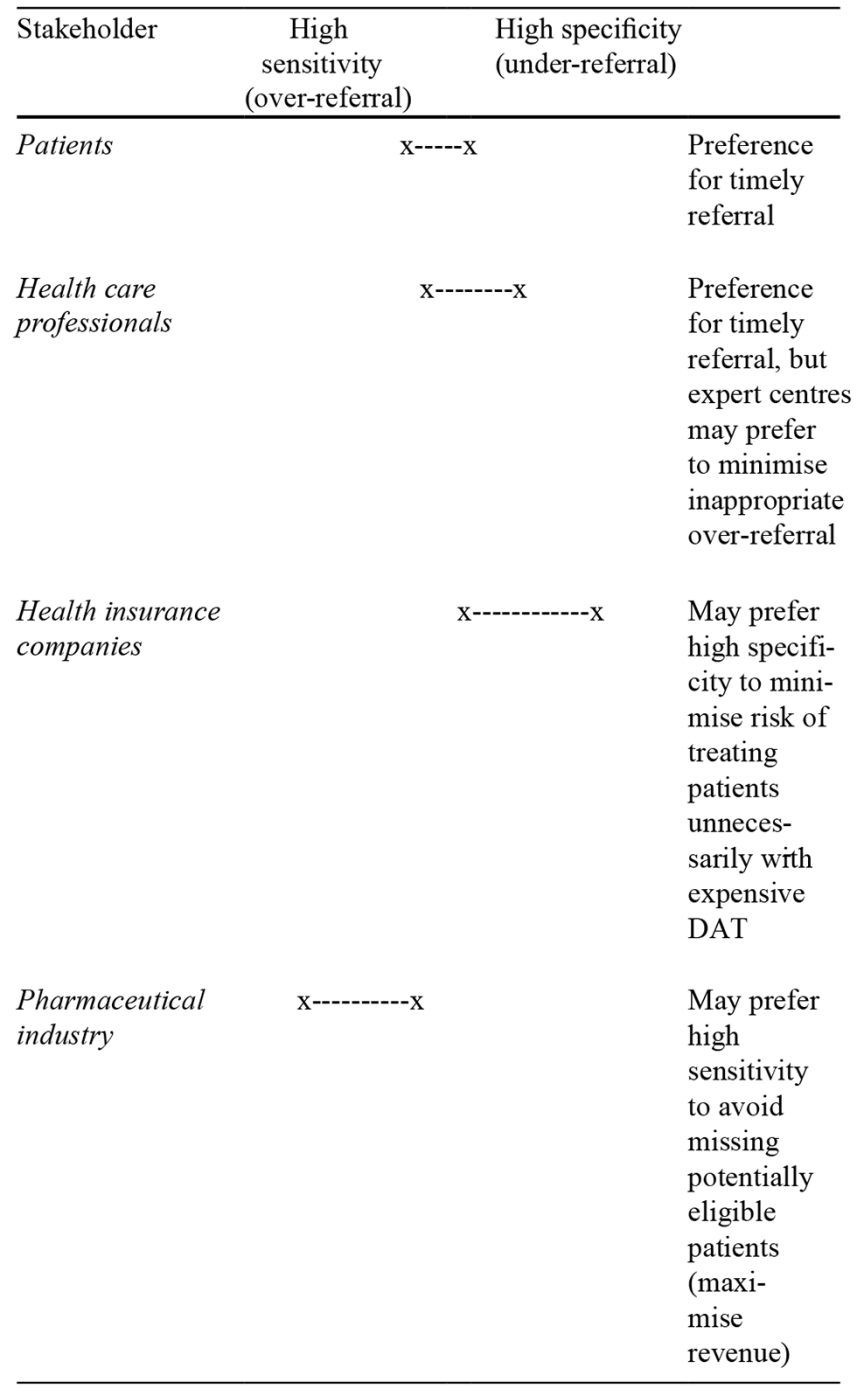
Possible stakeholder preferences regarding the balance between sensitivity and specificity of screening tools for referral for DAT

## Tools for timely referral for DAT

A review article from 2023 provides an overview of the available tools for timely referral for DAT (Moes et al., 2023b). Overall, there are tools for identifying patients with advanced PD, screening for eligibility for referral for *any* DAT, and screening for eligibility for DBS. A comprehensive review of the various tools is beyond the scope of this article.

In this article, we only consider the 5-2-1 criteria^1^ and D-DATS^2^, because these two criteria are the simplest and apply to all subtypes of DAT. Importantly, no uniform outcome measure was used in the development of the 5-2-1 criteria and D-DATS. Whereas the 5-2-1 criteria were developed for recognizing advanced PD, the outcome measure of D-DATS is eligibility for referral for DAT (Malaty et al. 2022; Moes et al. 2023d).

The screening performance of the two tools is reported in different studies (Aldred et al. 2020; Malaty et al. 2022; Moes et al. 2023a, d). However, only few studies have directly compared the performance of these tools. A study by Moes et al., in which the D-DATS was developed, compared the D-DATS with the 5-2-1 criteria (Moes et al. 2023d). Using decision curve analysis, this study demonstrated that D-DATS outperformed the 5-2-1 criteria at every possible cutoff point. At an arbitrarily chosen cutoff point of 6.4 points on the D-DATS, this tool had a higher specificity and positive predictive value than the 5-2-1 criteria, with almost similar sensitivity (Moes et al. 2023d).

The low specificity and low positive predictive value of the 5-2-1 criteria is also evident from the validation study by Malaty et al., although these figures were not explicitly reported in this study. These were calculated based on the data retrieved from this paper (Malaty et al. 2022; Moes et al. 2023b). Despite the relatively low positive predictive value of the 5-2-1 criteria, its use is recommended by different stakeholders (Malaty et al. 2022; AbbVie Pro website for UK Healthcare Professionals 2023).

## Knowledge gaps

The research on screening tools for timely referral for DAT is subject to a number of limitations.

The first and most important limitation is that D-DATS and the 5-2-1 criteria have only been compared in the Netherlands in an outpatient setting, with five Dutch experts serving as the reference test (gold standard) for determining eligibility for referral for DAT. It is unknown whether the reported diagnostic accuracy measures are also generalizable to other countries and to hospitalised patients. After all, there may be relevant international differences in the availability of resources, and the criteria for initiating DAT may also vary (Auffret et al. 2023). The latter point is supported by the OBSERVE-PD studies, which show considerable practice variation across European countries (de Oliveira et al. 2019; Takáts et al. 2020; Szasz et al. 2021; Pedrosa et al. 2022; Stefani et al. 2022; Fasano et al. 2022).

A second limitation is that current research on screening tools for DAT has been limited to reporting measures of screening performance (such as accuracy, sensitivity, specificity, positive predictive value, and area under the curve (AUC)). No empirical data have been presented on the relative ‘cost’ of a false-positive versus a false-negative classification. Consequently, there is no direct evidence that the use of these tools leads to better patient outcomes at affordable costs. This calls for further research with longer-term follow-up and comparisons of different tools.

Third, both the 5-2-1 criteria and D-DATS have a low sensitivity for identifying patients with treatment-resistant tremor or early-onset motor complications who may be candidates for DBS (Deuschl et al. 2022). In addition, the tools do not take into account activities of daily living or quality of life, both of which may be important in assessing eligibility for DAT.

A fourth limitation is the lack of research on the implementation and usability of the tools. Issues that need to be investigated include whether the tools are used consistently (e.g., ease of use, time constraints), whether the qualification of the user affects the outcome of the tool (e.g., differences between GPs, PD nurses and neurologists), and how often users override the tool’s recommendation.

## Impact analysis of clinical decision rules

The scientific literature on decision rules shows that clinical decision rules may help physicians when decisions are complex, when the stakes are high, or when there are opportunities to reduce costs without compromising patient care (McGinn et al. 2000; Reilly and Evans 2006).

**Fig. 2:**
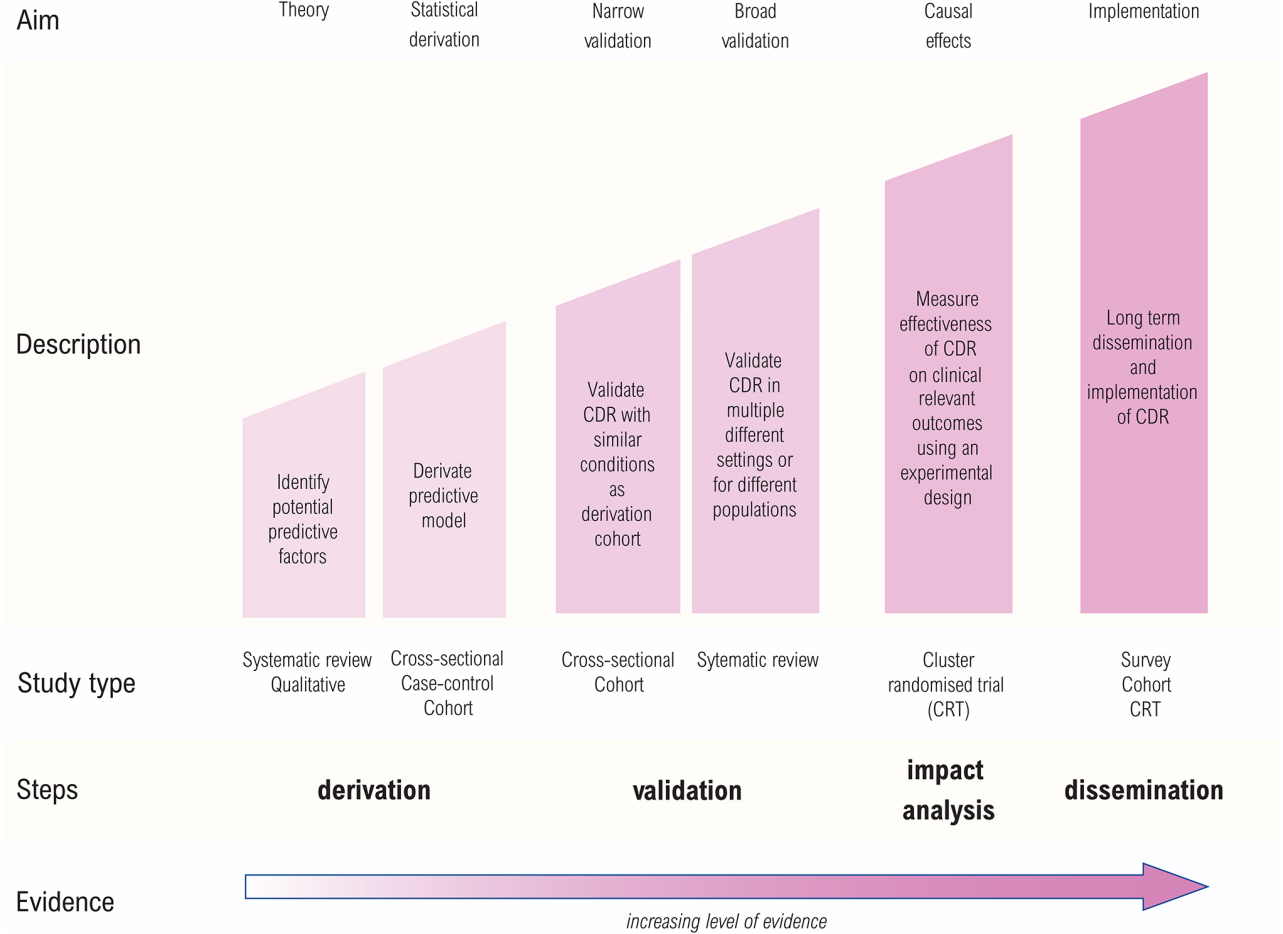
Development stages of a clinical decision rule (CDR). Figure adapted from Wallace et al. (Wallace et al. 2011). This figure is used under the terms of the Creative Commons Attribution License (https://creativecommons.org/licenses/by/2.0/)

Three excellent reviews on the development and evaluation of clinical decision rules have been written by Reilly & Evans, Wallace et al., and Cowley et al. ((Reilly and Evans 2006; Wallace et al. 2011; Cowley et al. 2019). These reviews and the underlying scientific literature distinguish three main phases in the development of a clinical decision rule: (1) derivation/development, (2) external validation, and (3) impact analysis to determine the impact on patient care. After the impact analysis, two additional phases can be considered: (4) cost-effectiveness analysis and (5) long-term implementation and dissemination (Fig. 2).

To maximise the accuracy and clinical utility of a clinical decision rule, it is important that it is developed, validated and evaluated in a systematic manner (Wallace et al. 2011). Reilly et al. argue that well-developed decision rules are exclusively evidence-based, their predictions empirically validated and their benefits proven in clinical trials (Reilly and Evans 2006). However, multiple review studies have demonstrated deficiencies in the methodological quality and reporting of development and validation studies, limiting the usefulness of clinical decision rules in practice (Bouwmeester et al. 2012; Collins et al. 2014; Cowley et al. 2019). To improve the quality of the development and validation of clinical decision rules, it is recommended to use the widely accepted TRIPOD (Transparent Reporting of a multivariable prediction model for Individual Prognosis or Diagnosis) guidelines (Collins et al. 2015).

The optimal study design for an impact analysis is a cluster randomised trial with centres as clusters (Wallace et al. 2011; Cowley et al. 2019). However, cluster randomised trials are associated with significant practical, logistical, and economic challenges (Cowley et al. 2019). Therefore, evaluating the impact of a decision rule has been described as ‘the next painful step’ in the development process (Lee 1990).

## Intended research

Does a model that has been shown in validation studies to adequately predict eligibility for DAT referral still require an impact analysis using a large, multicentre, cluster-randomised trial? The answer to this question depends on the number of acceptable false positive and false negative predictions and their impact on patient management and outcomes (Moons et al. 2009). In the absence of empirical data, further research is required to determine which of the two screening tools is better (5-2-1 criteria or D-DATS) and whether implementation of these tools leads to a demonstrably beneficial effect (quality of life, cost-effectiveness).

To address the identified knowledge gaps, we propose to conduct three studies. The first two studies will focus on the broad external validation of the D-DATS and the 5-2-1 criteria. The third study is an impact analysis of both tools.

In order to broaden the external validation, we will start by extending the D-DATS validation study (referred to as VALIDATE) to an evaluation by European experts and possibly also international experts outside of Europe (Moes et al. 2023a). To this end, we will invite a number of international PD experts to assess a random selection of patient vignettes from VALIDATE. As in the VALIDATE-study, the initial assessment of eligibility for referral for DAT will be made individually by each expert. Once the individual assessments of the international experts have been collected, the experts will meet to discuss cases of disagreement. Ultimately, the judgment of the expert panel will be determined by group consensus. The group consensus judgment of this international panel of experts is then used to validate D-DATS and compare it with the 5-2-1 criteria.

As a second study, we plan to perform a retrospective analysis of consecutive patients referred for DAT in several international expert clinics. We will examine the ability of the tools to triage referrals, i.e. to assess the appropriateness of actual referrals. It should be noted that in a retrospective study design, sensitivity and specificity cannot be investigated, as there are no data available for the non-referred patients.

**Fig. 3:**
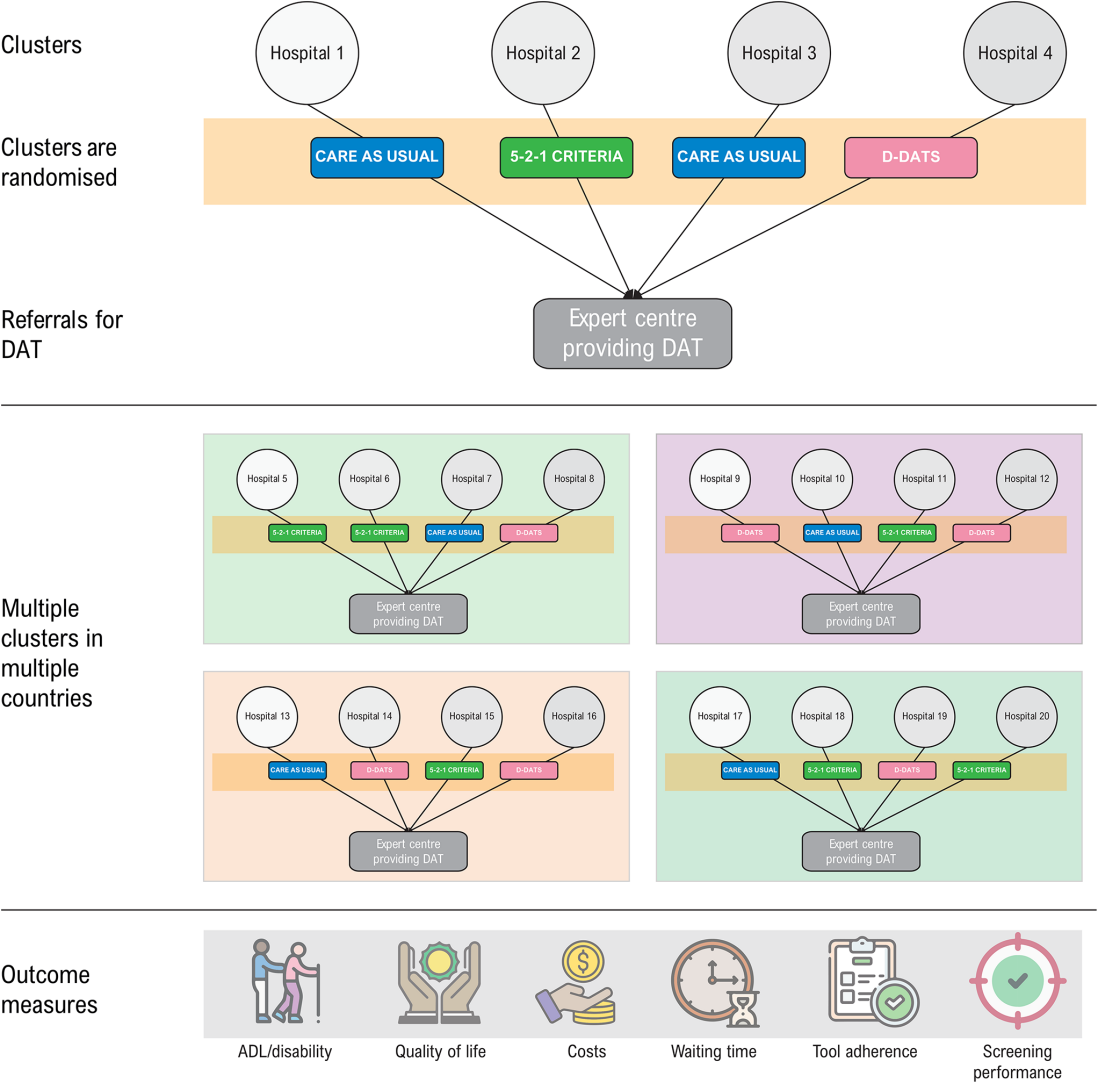
Draft design for a cluster randomised trial to analyse the impact of screening tools on the timely referral of people with Parkinson’s disease for a device-aided therapy (DAT)

Thirdly, to examine the impact of the two clinical decision rules (D-DATS and 5-2-1 criteria), a cluster randomised controlled trial (RCT) needs to be conducted (Fig. 3). In this study, the primary research question is whether implementation of D-DATS improves quality of life outcomes for patients with PD. The secondary research question is whether D-DATS outperforms the 5-2-1 criteria in reducing unnecessary referrals and improving outcomes. The cluster RCT comprises three groups: a group receiving care as usual (no tool), a group in which referral is based on D-DATS, and a group in which referral is based on the 5-2-1 criteria. The primary outcome measure will be patient quality of life (e.g., PDQ-8 (Jenkinson et al. 1997), and the secondary outcome measures are disability (e.g., MDS-UPDRS part II, or the AMC Linear Disability Score (ALDS) (Weisscher et al. 2007; Odekerken et al. 2013; Rodriguez-Blazquez et al. 2013; Gonzalez-Robles et al. 2023)), the number of patients receiving DAT, the number of early referrals, and the number of medication adjustments at the expert centre.

The proposed multinational cluster RCT will be conducted in several European countries. In each participating country, there will be one expert centre that already receives a high volume of referrals for DAT from general clinics in the vicinity of the expert centre. Depending on the exact study design, two to five referral centres per expert centre will participate. Randomisation per referring clinic will be used for tool assignment (no tool [care as usual], 5-2-1 criteria, or D-DATS). In order to ensure the generalisability of the study results, a multinational design will be employed, which will facilitate the enrolment process and allow the required sample size to be reached in a timely manner.

## Conclusion

Given the increasing prevalence of Parkinson’s disease (PD), the growing need to efficiently utilise healthcare resources and minimise costs, and the recommendation that eligible PD patients should be offered treatment with DAT, it is of critical importance to implement an accurate screening tool for timely referral for DAT. Although several tools are available aiming to achieve this goal, it is still unclear which tool performs best. Furthermore, it remains unclear whether widespread implementation of the optimal screening tool will result in improvements in quality of care and cost-effectiveness. To address these pressing questions, international collaboration in methodologically sound research is essential.

## Footnotes

1 The 5-2-1 criteria are similar to MANAGE-PD, but MANAGE-PD is more comprehensive. MANAGE-PD and the 5-2-1 criteria are based on a Delphi study sponsored by AbbVie (Antonini et al. 2018). It is important to note that these tools have not been developed in accordance with accepted guidelines for decision rule development (e.g., TRIPOD guideline (Collins et al. 2015; Cowley et al. 2019).

2 The development study of D-DATS followed the recommendations of the TRIPOD guideline. In the development study by Moes et al. 2023d, the tool had not been given a specific name. However, the tool is now recognised as D-DATS, an acronym that stands for Dutch Device-Aided Therapy Screening. This acronym was first used in the review by Moes et al. 2023b. The adjective “Dutch” refers to the country in which the tool was developed and validated, but it should be noted that the tool is in fact available in English.
